# The cellular and molecular mechanisms of vertebrate lens development

**DOI:** 10.1242/dev.107953

**Published:** 2014-12

**Authors:** Aleš Cvekl, Ruth Ashery-Padan

**Affiliations:** 1 Department of Genetics, Albert Einstein College of Medicine, Bronx, NY 10461, USA; 2 Department of Ophthalmology and Visual Sciences, Albert Einstein College of Medicine, Bronx, NY 10461, USA; 3 Sackler School of Medicine and Sagol School of Neuroscience, Tel-Aviv University, 69978 Ramat Aviv, Tel Aviv, Israel

**Keywords:** Cell determination, Crystallins, Differentiation, Lens, Pax6, Pre-placodal region

## Abstract

The ocular lens is a model system for understanding important aspects of embryonic development, such as cell specification and the spatiotemporally controlled formation of a three-dimensional structure. The lens, which is characterized by transparency, refraction and elasticity, is composed of a bulk mass of fiber cells attached to a sheet of lens epithelium. Although lens induction has been studied for over 100 years, recent findings have revealed a myriad of extracellular signaling pathways and gene regulatory networks, integrated and executed by the transcription factor Pax6, that are required for lens formation in vertebrates. This Review summarizes recent progress in the field, emphasizing the interplay between the diverse regulatory mechanisms employed to form lens progenitor and precursor cells and highlighting novel opportunities to fill gaps in our understanding of lens tissue morphogenesis.

## Introduction

**Fig. 1.**
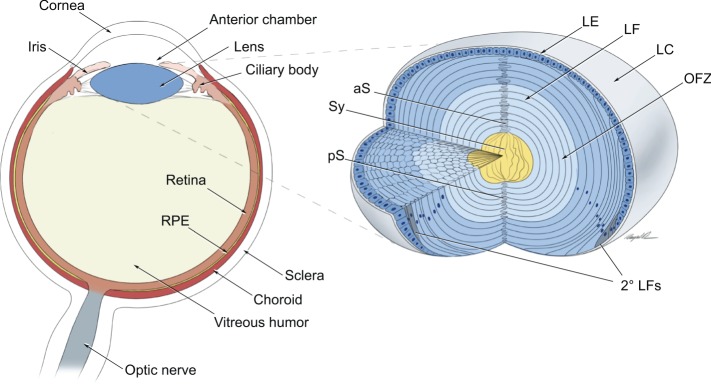
**The vertebrate eye and lens 3D structure.** Illustration of a transverse section of the vertebrate eye, showing the principal eye tissues and their arrangement within the eyeball. The lens, which is part of the anterior segment of the eye, consists of the lens epithelium and lens fibers, which make up the bulk of the lens mass. Newly formed lens fibers are deposited on the top of the ‘older' central cells in the form of concentric shells of hexagonally packed and radially aligned fiber cells. aS, anterior suture; LE, lens epithelium; LF, lens fibers; LC, lens capsule; OFZ, organelle-free zone; pS, posterior suture; 2° LFs, secondary lens fibers; Sy, syncytium. Adapted with permission from Shi et al. (2009).

The eye is a sensory organ that is assembled from tissues that can be described in functional biological/engineering terms as: optical (lens, cornea and iris), light sensing [photoreceptors shielded by retinal pigmented epithelium (RPE)], transmission (part of the retina and optic nerve) and display/memory card (visual cortex). The ocular lens differs from all other organs in that it is avascular without any innervation and is composed of one cell type that reaches different stages of differentiation and originates from a single common cell – the lens precursor cell. Lens cells are of ectodermal origin and ultimately differentiate into either lens fibers, which make up the bulk of the lens mass, or the lens epithelium, which is sheet of cuboidal epithelium that covers the anterior surface of the lens (Fig. 1). This single cell type origin combined with the relatively simple morphology of the lens makes it an advantageous model system with which to address many fundamental problems of cellular and developmental biology.

Over 100 years of lens studies in vertebrates have produced a remarkably complex picture of the inductive processes governing the formation and differentiation of lens cells. Findings from the last decade have provided novel insights into the cellular and molecular mechanisms of the entire cascade of lens embryonic induction and the dynamics of lens differentiation and morphogenesis. In addition, several hallmark lens-based studies have had major impacts on our general understanding of cell cycle regulation-coupled cellular differentiation and its dysregulation in cancer cells; from the establishment of the first mammalian tissue-specific promoter used in transgenic mice (Overbeek et al., 1985) that enabled molecular studies of tumor virus-induced oncogenesis *in vivo* (Mahon et al., 1987), to lens-based studies of the retinoblastoma 1 (*Rb1*, or *pRb*) and *p53* (*Tp53*, or *Trp53*) tumor suppressor genes (Morgenbesser et al., 1994; Pan and Griep, 1994, 1995; Nakamura et al., 1995) and of negative regulators of cell cycle progression, such as p27^Kip1^ (Cdkn1b) and p57^Kip2^ (Cdkn1c) (Zhang et al., 1998). More recently, novel experimental systems based on the differentiation of human embryonic stem cells (ESCs) have significantly increased our repertoire of experimental models for studying lens formation (Yang et al., 2010; Dincer et al., 2013; Leung et al., 2013), and disease-specific model systems of lens developmental abnormalities, using induced pluripotent stem cells (iPSCs), are currently being developed.

This Review provides an up-to-date summary of the cellular and molecular mechanisms that regulate the various stages of lens development, beginning with those that are employed in the formation of lens progenitor cells. We further discuss the formation of the lens placode and its invagination to form the lens vesicle, cell cycle regulation in lens cells, and the specific factors and processes involved in lens fiber cell differentiation.

## The embryological origin of the lens: an overview

Lens development (see Piatigorsky, 1981) is a key event during eye organogenesis, and abnormal lens development results in a range of lens structural abnormalities and cataract formation (see Box 1). Lens morphogenesis is first manifested as a thickening of the head surface ectoderm to form the lens placodes, which are composed of lens progenitor cells that display a palisade-like morphology. The invagination of these cells establishes the initial lens 3D structure, the lens vesicle, which is composed of lens precursor cells. The anterior cells of the lens vesicle give rise to the lens epithelium, while the posterior lens vesicle cells elongate and produce the primary lens fibers that form the embryonic lens nucleus. The proliferation of lens epithelial cells produces new rows of cells that, upon cell cycle exit, generate secondary lens fiber cells, which form the outer shells of the lens and contribute to lens growth throughout life (Fig. 1).

Box 1. **Abnormal lens development: cataracts, amblyopia, presbyopia and ageing**

Disrupted lens microarchitecture can result in lens opacification and cataracts. Congenital cataracts are typically caused by mutations in genes encoding lens regulatory (e.g. FOXE3, HSF4, MAF, PAX6 and PITX3) and structural (e.g. crystallins, connexins, MIP, BFSP2, EPHA2, EPHA5 and LIM2) proteins (see Shiels and Hejtmancik, 2013). It is likely that mutations in these genes also play roles in the more commonly occurring age-onset cataracts, either directly and/or as gene modifiers. Age-onset cataract is a common disease in individuals over age 65 and the most common form of reversible blindness (see Petrash, 2013). Although congenital cataracts are rare (3-4 incidences per 10,000 newborns), they require immediate removal through lens replacement in order to allow normal visual development and prevent amblyopia (‘lazy eye’), and identification of the underlying mutations is important for genetic counseling. Unlike most organs, the lens grows throughout the lifespan of the organism. Increased size of the lens, coupled with reduced elasticity, changes its accommodative power to near objects, resulting in age-related presbyopia. Inhibition of cell growth through modulating secondary lens fiber cell differentiation might provide a strategy to delay the onset and/or slow down the progression of this common visual impairment.

At the cellular level, an interplay of extracellular signaling, commonly referred to as inductive processes, dictate cell fate decisions (Box 2). At the molecular level, cell type identity is determined by a specific combination of local activators and signal-regulated DNA-binding transcription factors (see Barolo and Posakony, 2002) assisted by a range of chromatin remodeling enzymes (see Cvekl and Mitton, 2010). Thus, studies of DNA-binding transcription factors expressed throughout lens formation and their connectivity with extracellular signaling have provided many important insights into lens formation.

Box 2. **Cell fate determinants: competence, specification and commitment/determination**

The specificity of cell signaling is achieved through a combinatorial principle that involves local signal concentration, time of exposure, and the presence/absence of inhibitory signaling molecules. The crucial factor is the ‘competence’ of individual cells to interpret these signals. Competence requires the presence of specific transmembrane receptors on the ‘induced’ cell surface, as well as the existence of cytoplasmic/nuclear machinery to relay and interpret these signals. The inductive process can be operationally divided into a ‘specification’ and a subsequent ‘commitment’ phase. Tissue specification is characterized by a stable cell fate in a neutral medium. Cells are committed if they do not change their cell fate regardless of their environment (see Patthey and Gunhaga, 2014).

Combining both cellular and molecular levels, lens morphogenesis can be divided into at least four general phases. In the initial phase, a novel cell type is established from multipotent placodal precursors and these cells are induced to form the lens placode through a Pax6/Six3-dependent gene regulatory network (GRN) in conjunction with paracrine (from the prospective retina) and autocrine bone morphogenetic protein (BMP) signals, paracrine retinoic acid (RA) signaling and inhibition of Wnt signaling in the presumptive lens ectoderm. In the second phase, the lens placode, which is composed of elongated cells, invaginates to form the lens pit and lens vesicle, which is the initial lens 3D structure. The third stage of lens formation involves the initiation of primary lens fiber cell differentiation by tightly controlled cell cycle exit regulated by BMP, fibroblast growth factor (FGF) and Notch signaling, as well as lens epithelium differentiation. The fourth and final phase is a lens fiber cell ‘engineering' process. During this time, the correct mechanical stiffness of the lens, which is required for light focus and accommodation and requires a modified cytoskeleton, is established. In addition, the degradation of subcellular organelles, which is required for lens transparency, and tissue remodeling are mechanistically linked to a tightly controlled proteolytic apparatus, while the materials needed to build the lens fiber cell are made prior to the cessation of protein synthesis.

In the following sections, we provide a detailed summary of the cellular and molecular steps involved in each of these phases of lens development, from the initial induction of the lens ectoderm to the final stages of lens fiber maturation.

## The initial phase: partitioning of the neural plate border and the specification of prospective lens cells

**Fig. 2.**
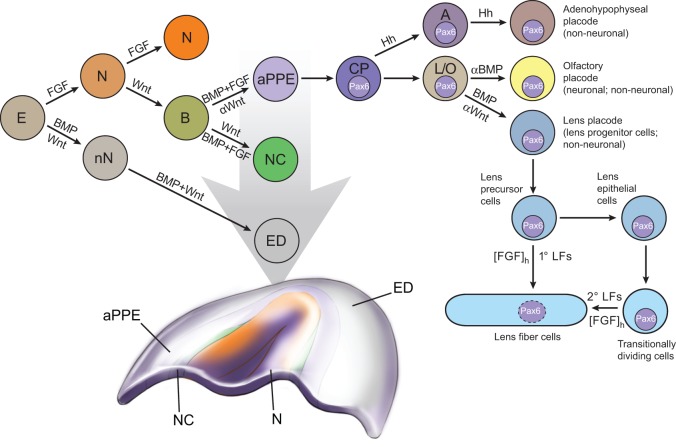
**Cell fate decisions during lens induction.** Cell fate decisions and differentiation steps that occur prior to and during lens induction and differentiation. The signaling pathways involved (BMP, FGF and Wnt) as well as the inhibition of specific pathways (indicated by αBMP and αWnt) are shown (see Patthey and Gunhaga, 2014). Briefly, the induction process involves sequential partitioning of the anterior ectoderm (E) into neural ectoderm (N), non-neural ectoderm (nN), border ectoderm (B), anterior pre-placodal ectoderm (aPPE), neural crest (NC) cells, epidermis (ED), Pax6^+^ common progenitors (CP), adenohypophyseal progenitors (A) and, finally, a common lens-olfactory progenitor (L/O). The inductive events culminate with the formation of Pax6^+^ common progenitor cells within the area of the anterior pre-placodal ectoderm. The common pre-placodal progenitors are thus multipotent cells that ultimately produce distinct neuronal and non-neuronal cell types, as shown by the reiterative use of BMP signaling for non-neuronal lens formation and its transitional inhibition (αBMP) required for the formation of the neurogenic olfactory placode (see text for details). In addition to the contribution of BMP/FGF/Wnt signaling, a recent study revealed a surprising role for the neuropeptides somatostatin and nociceptin, emanating from the anterior mesendoderm, in controlling lens and olfactory placode development in chick and fish embryos by promoting Pax6 expression. This intriguing finding implicates ancestral roles for neuropeptides in patterning of the embryo, prior to their functions in the mature nervous and endocrine systems (Lleras-Forero et al., 2013). 1° and 2° LFs, primary and secondary lens fibers; [FGF]_h_, high concentration (>30 ng/ml) of FGF2.

Following neural plate formation (Munoz-Sanjuan and Brivanlou, 2002), the ectoderm of the vertebrate embryo is divided into three domains: non-neural ectoderm (prospective epidermis); the neural plate; and a third region, known as the ‘border', which lies between the neural plate and the non-neural ectoderm (Fig. 2) (Litsiou et al., 2005; Grocott et al., 2012). The anterior part of the neural plate border gives rise to the anterior pre-placodal region (aPPR) (Litsiou et al., 2005), while in the more posterior regions neural crest cells are also formed from the border ectoderm (Fig. 2). The formation of the aPPR, through active FGF and BMP signaling combined with inhibition of Wnt signaling, has been reviewed in detail elsewhere (McCabe and Bronner-Fraser, 2009; Patthey and Gunhaga, 2014). The anterior pre-placodal cells give rise to the individual placodal progenitors that migrate and converge into the individual placodes: adenohypophyseal, olfactory, and lens (Fig. 2). Evidence exists that a common progenitor cell can give rise to the lens as well as olfactory placodal cells (Sjodal et al., 2007). Taken together, studies suggest that pre-placodal cells are specified at a single-cell level within a field of cells rather than from a discrete region of the surface ectoderm as previously implicated by ‘classical' embryological studies (see Grainger, 1992).

The identification of pre-placodal cells by cell-tracing experiments was conducted in chick (Bhattacharyya et al., 2004) and zebrafish (Whitlock and Westerfield, 2000) but has not yet been carried out in mouse embryos, although the identification of regionally specific DNA-binding transcription factors expressed within the mouse putative aPPR supports similar cellular mechanisms among the model organisms. In mice, the expression of Foxg1 (Duggan et al., 2008), Otx2 (Steventon et al., 2012) and Six3 (Liu et al., 2006) was shown to occur from embryonic day (E) 8 (the 1-7 somite stage), and this was followed by the onset of Pax6 expression in a broad region of the head surface ectoderm (Walther and Gruss, 1991). However, the expression of the *Foxg1*, *Otx2* and *Six3* ‘early' genes is not specific for this region, and somatic knockouts of these genes produce a series of abnormalities due to their broader expression domains in the neural plate and elsewhere (Xuan et al., 1995; Acampora et al., 1999; Lagutin et al., 2003). Pax6 is expressed in the region of anterior surface ectoderm corresponding to the future adenohypophyseal, olfactory and lens placodes as well as in the optic vesicle (OV) and other parts of the future brain. Importantly, *Pax6* null mice do not form olfactory and lens placodes (Quinn et al., 1996; Ashery-Padan et al., 2000) and show disrupted pituitary gland development (Kioussi et al., 1999). These data support the idea that the most anterior placodes evolve from a common cell progenitor (Fig. 2) that is marked by, and is dependent on, Pax6 expression (Bailey et al., 2006; Sjodal et al., 2007). Interestingly, to date, no single gene with a specific pre-placodal expression domain and/or function has been identified, possibly reflecting the transient state of the pre-placodal cells, which share molecular features with other embryonic progenitors.

## Completion of the initial phase: lens placode formation and initiation of the lens differentiation program

**Fig. 3.**
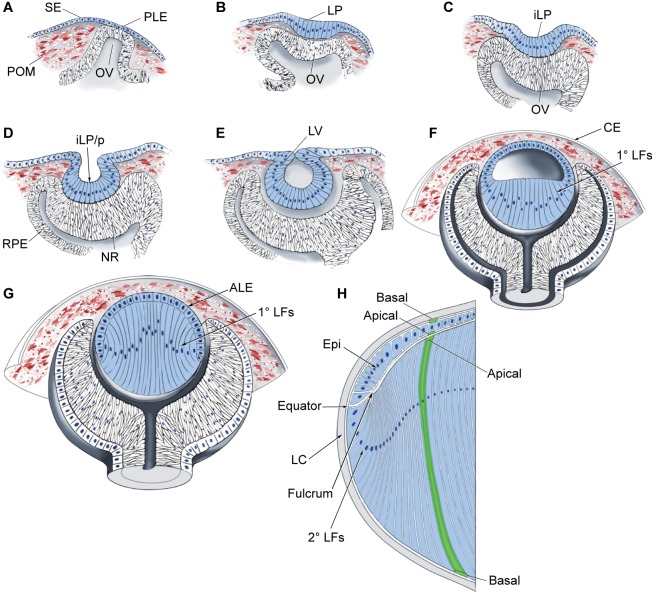
**Stages of lens formation in mouse embryos.** Schematics showing the stages of lens development at various points during mouse embryogenesis. (A) E9.0, prospective lens ectoderm. (B) E9.5, lens placode. (C) E10, invaginating lens placode. (D) E10.5, invaginating lens placode to lens pit. (E) E11, open lens vesicle. (F) E12.5, primary lens fiber cell differentiation. (G) E13.5-E14.5, completion of primary lens fiber cell elongation to secondary lens fiber cell formation. (H) Lens growth and secondary lens fiber cell differentiation in adult ocular lens. The apical-basal polarity of lens epithelial and fiber cells is indicated. The area where the apical tips of elongating epithelial cells at the equator constrict to form an anchor point before fiber cell differentiation and elongation at the equator was recently named the ‘lens fulcrum' (Sugiyama et al., 2009). ALE, anterior lens epithelium; CE, corneal epithelium; iLP, invaginating lens placode; iLP/p, invaginating lens placode/lens pit; LC, lens capsule; Epi, lens epithelium; LP, lens placode; NR, neuroretina; OV, optic vesicle; POM, periocular mesenchyme; 1° and 2° LFs, primary and secondary lens fibers; PLE, prospective lens ectoderm; RPE, retinal pigmented epithelium; SE, surface ectoderm.

The transition from the prospective lens ectoderm (PLE) to the lens placode involves cell-tissue interactions, including those involving the surrounding periocular mesenchyme (POM) and the underlying OV (Fig. 3). Chick anterior pre-placodal cells, when grown in isolation, acquire lens-forming competence (Bailey et al., 2006). *In vivo*, however, lens placodes are formed only in restricted domains overlaying the OV. This regional restriction is assured by the active inhibition of lens fate by the POM surrounding the OV via the expression of TGFβ ligands that induce both Smad3 and Wnt/β-catenin activity and inhibit Pax6 expression in the non-lens ectoderm (Grocott et al., 2012). The importance of inhibitory cues for proper localization of the lens in mouse is further supported by gain- and loss-of-function studies of β-catenin activity in the PLE; activation of canonical Wnt/β-catenin signaling inhibits lens formation (Smith et al., 2005), whereas loss of β-catenin induces ectopic lentoid formation in the periocular ectoderm (Kreslova et al., 2007). Furthermore, although the transcriptional co-activator and nuclear protein pygopus 2 (Pygo2), which is expressed in both the lens placode and POM and influences lens formation, is a target of Wnt signaling in the lens, its role in lens induction, through activation of Pax6 expression, is Wnt independent (Song et al., 2007). Future studies should aim to determine the contribution of the ocular mesenchyme, as well as of TGFβ signaling, to the inhibition of lens fate and the alignment of eye structures in mammals.

The OV plays dual roles in lens placode formation: it functions as a physical barrier to prevent inhibitory signals from the POM from reaching the PLE (‘permissive' role) and it may generate signals directed towards the PLE (‘instructive' role). For example, selective depletion of Pax6 in the OV disrupts lens placode formation (Klimova and Kozmik, 2014); however, Pax6-dependent genes that function in this process remain elusive. Other key components of the lens-inducing cellular machinery include BMP and RA signaling. In the mouse embryo, depletion of BMP4, which is normally expressed abundantly in the OV and less strongly in the surface ectoderm and POM, completely blocked the lens induction process even though the expression of Pax6 and Six3 in the PLE was not reduced (Furuta and Hogan, 1998). A readout of BMP4 signaling in the PLE is expression of the transcription factor Sox2, the ‘basal' levels of which are not enhanced in *Bmp4^−/−^* embryos (Furuta and Hogan, 1998). Interestingly, exogenous BMP4 could not rescue lens formation in *Bmp4^−/−^* embryos in the absence of a lens vesicle (Furuta and Hogan, 1998), which points to additional signaling that functions alongside BMP4, as well as emphasizing the OV barrier role described above.

In contrast to the role of BMP4, the role of BMP7 in the early stages of lens formation remains to be clarified. Initial studies of *Bmp7*-deficient embryos showed panocular defects linked to defective lens induction based on the loss of Pax6 expression in the PLE (Luo et al., 1995; Wawersik et al., 1999). In subsequent studies, the majority of *Bmp7^−/−^* embryos initiated normal lens development (Morcillo et al., 2006). This variable penetrance probably reflects differences in the genetic backgrounds of the mice analyzed and also implicates BMP4 as the essential activator of lens induction. Another possibility is that the expression domains of BMP7 change rapidly between E9.5 and E11 (Wawersik et al., 1999), and this might be linked to the variable penetrance of the eye abnormalities found in each mouse strain. Consistent with the data on BMPs produced by the early embryonic eye, conditional inactivation of the type I BMP receptors *Bmpr1a* and *Acvr1* in the PLE also disrupted the early stages of lens development and demonstrated that BMP receptor signaling also regulates both cell survival and proliferation during lens placode formation (Rajagopal et al., 2009).

**Fig. 4.**
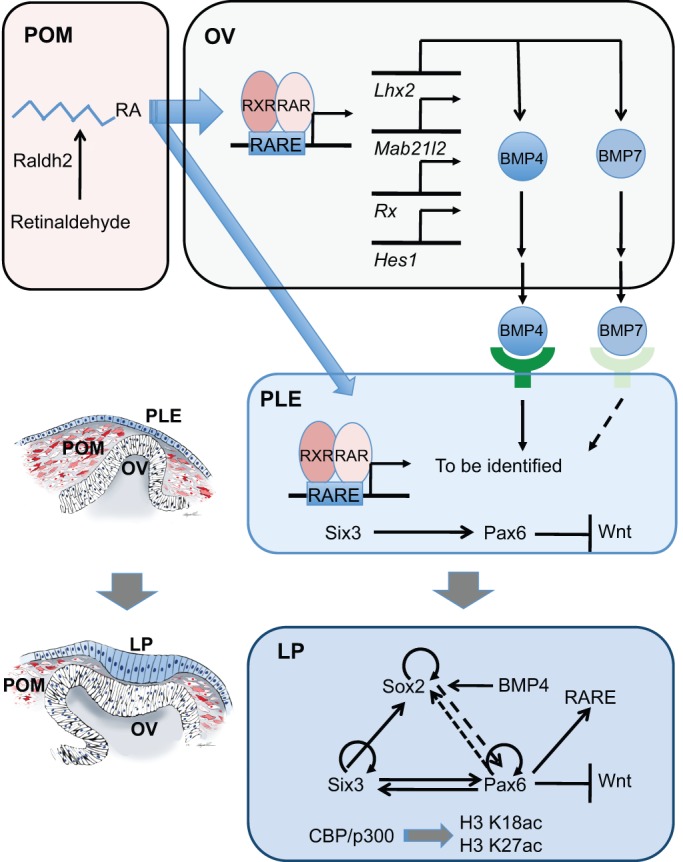
**A combination of molecular and cellular mechanisms underlies the transition from prospective lens ectoderm to lens placode.** At E8.5, paracrine BMP and retinoic acid (RA) signaling from the optic vesicle (OV) influences the prospective lens ectoderm (PLE). The expression of BMP4 and BMP7 in the OV is regulated by the *Lhx2* homeobox gene. The OV, in order to form normally and support lens placode formation, requires the cell-autonomous functions of multiple genes, including *Hes1*, *Lhx2*, *Mab21l1*, *Pax6* and *Rx*. In addition, RA is synthesized from retinol (vitamin A) by retinol dehydrogenase Rdh10 into retinaldehyde that is converted into RA by the enzyme Aldh1a2 (Raldh2) expressed in the periocular mesenchyme (POM). In the PLE, expression of Pax6 is activated by Six3 and possibly additional transcription factors. RA signaling is active from E8.75 in the PLE. BMP and RA target genes in the PLE remain to be identified. In the lens placode (LP), the Pax6, Six3 and Sox2 GRN is active. Specific modules of this GRN govern lens placode formation (Pax6/Six3) and its subsequent invagination (Pax6/Six3/Sox2). The regulatory interactions between Pax6 and Sox2 are dynamic and change over time (dashed line). It is notable that Pax6 is also required for RA signaling in the PLE (Enwright and Grainger, 2000), and for suppression of Wnt signaling (Kreslova et al., 2007). The CBP and p300 proteins acetylate residues K18 and K27 of histone H3. These acetylations are thought to be required for proper expression of the Six3/Pax6/Sox2 GRN within the lens placode. It is also noteworthy that species-specific differences may exist. For example, Notch signaling is not necessary for lens placode formation in mouse (Le et al., 2012), but is important in the frog model (Ogino et al., 2008). RARE, retinoic acid response element.

Studies of multiple genes linked to BMP signaling further corroborate the central role of this signaling pathway in lens placode induction. For example, knockout of the transcription factor *Lhx2*, which is expressed only in the OV, inactivated Pax6 expression in the PLE but not in the OV (Porter et al., 1997). Notably, *Lhx2^−/−^* OVs do not express BMP4 and BMP7 (Yun et al., 2009). Inactivation of *Mab21l2*, which is highly expressed in the OV and is thought to function in BMP signaling, is also detrimental for lens placode formation (Yamada et al., 2004). Lens placode formation also requires the transcription factors Rx (Rax) (Mathers et al., 1997) and Hes1 (Lee et al., 2005), both of which are expressed in the OV; however, their possible links to BMP signaling remain to be established (Fig. 4). These results are consistent with a paracrine role for BMP signaling in lens placode formation and normal OV development, as described above. Conversely, recent studies have shown that surface ectoderm-derived BMP and Wnt signals are used to specify the RPE within the prospective optic cup (Steinfeld et al., 2013).

RA signaling also plays a multitude of roles during eye development, including lens induction (see Cvekl and Wang, 2009). At the beginning of eye development, RA is generated by Aldh1a2 (Raldh2) enzymes within the temporal POM that is in contact with the OV (Fig. 4). RA signaling is active in the OV at E8.5, but not in the surface ectoderm, where its activity is first detected from E8.75 and continues throughout the subsequent stages of lens placode formation and invagination. Taken together, paracrine RA signaling is required for the reciprocal invagination of the lens pit/optic cup (Mic et al., 2004; Molotkov et al., 2006).

It had been suggested that paracrine FGF signaling from the OV also functions to regulate lens induction; however, conditional inactivation of the FGF receptors *Fgfr1* and *Fgfr2* in the PLE did not prevent placode formation, although the mutated cells exhibited diminished survival (Garcia et al., 2011). Increased cell death within the lens placode was also found in *Fgfr2/Bmpr1a* compound mutants (Garcia et al., 2011). Collectively, paracrine/autocrine BMP signaling and paracrine RA signaling, combined with the tightly controlled expression of Pax6, Six3 and Sox2 in the PLE, establish an intricate system that governs lens placode formation. Additional studies are required for a more complete understanding of this process.

## Lens placode formation: the ‘core’ lens GRN

Two decades of molecular studies have focused on deciphering the regulatory interactions between Pax6, Six3 and Sox2, and the hierarchy of this regulation, via the identification of their promoters, enhancers and target genes during lens morphogenesis. In the current model, Six3 regulates the onset of Pax6 expression in the PLE from E8.0 (Goudreau et al., 2002; Liu et al., 2006) (Fig. 4). Subsequently, Pax6 regulates expression of Six3 in PLE (Goudreau et al., 2002; Purcell et al., 2005; Liu et al., 2006). By contrast, pre-placodal Pax6 expression does not require Sox2; however, Pax6 regulates Sox2 expression in the lens placode (Smith et al., 2009). From E8.75, Pax6 expression is controlled by an evolutionarily conserved ectodermal enhancer (EE) (Dimanlig et al., 2001), which contains binding sites for Meis1/2, Oct1 (Pou2f1), Pax6, Pknox1, Six3 and Sox1/2 (Aota et al., 2003; Zhang et al., 2002; Liu et al., 2006; Donner et al., 2007; Rowan et al., 2010). The autoregulation of Pax6 is a positive feedback mechanism that ensures maintenance of cell type identity following cell divisions (Ptashne, 2013). Furthermore, both Six3 (Liu et al., 2006) and Sox2 (Inoue et al., 2007) also autoregulate their expression (Fig. 4) although the expression of Sox2 is abolished following the completion of lens placode invagination. Nevertheless, Pax6 and Sox2 jointly regulate downstream targets, including δ-crystallin in chick (Kamachi et al., 2001) and N-cadherin in mice (Smith et al., 2009), which is required for detachment of the lens vesicle from the surface ectoderm (Pontoriero et al., 2009). In addition, a Pax6-Sox2 binary complex functions as a powerful transcriptional activator (Kamachi et al., 2001; Inoue et al., 2007).

The gradual acquisition of lens cell fate and the dynamic transition in the regulatory interactions between the DNA-binding transcription factors described above are tightly linked to chromatin structure and dynamics. Recent functional studies of chromatin modifiers during lens development support this notion. For example, the transition from PLE to lens placode requires the histone acetyltransferases CBP (Crebbp, or Kat3a) and p300 (Kat3b), as their inactivation in the PLE results in the attenuation but not elimination of Pax6, Six3 and Sox2 expression, while the expression of Foxe3, Prox1 and crystallin genes was not initiated (Wolf et al., 2013b). Given the prominent roles of CBP/p300 enzymes as crucial enhancer-binding proteins (Visel et al., 2009), depletion of these proteins in *CBP^−/−^; p300^−/−^* mutated ectoderm probably leads to disfunction of lens-specific enhancers, including those in *Pax6*, *Six3* and *Sox2* loci. Accordingly, Pygo2 (Song et al., 2007) contains a PHD domain that recognizes methylated histones and these modifications may cooperate with acetylated histones, catalyzed by CBP/p300, to regulate *Pax6* transcriptional enhancers.

## The second phase: invagination of the lens placode and lens vesicle formation

A series of recent studies has provided novel insights into the molecular and cellular mechanisms that mediate epithelial bending during lens placode transition into the lens pit. This morphological transition depends on a combination of processes, including cell proliferation, cell crowding and cytoskeletal reorganizations. Moreover, as lens placode invagination is synchronized with OV invagination to form the optic cup, the interplay between the structures needs to be taken into consideration as an important mechanism of lens pit morphogenesis.

Cell proliferation within the placode increases its size (Fig. 3A,B) and adhesion leads to cell crowding and is followed by placode invagination (Fig. 3C,D). Lens progenitor cell proliferation is regulated by neurofibromatosis 1 (*Nf1*), which encodes a small Ras GTPase-like protein (Carbe and Zhang, 2011). In *Nf1^−/−^* embryos, the lens placode is reduced in size due to deficient cell proliferation. Consequently, the lens pit is also smaller and does not form the lens vesicle. The subsequent stages of placode invagination involve a number of cellular changes, including changes to the extracellular matrix (ECM) and cytoskeleton. The accumulation of ECM between the PLE and the OV prevents the prospective lens cells from spreading, and this process is dependent on ectodermal expression of Pax6, which in turn directly or indirectly regulates the expression of multiple ECM proteins, including fibronectin 1 (Fn1), versican (Vcan, or Cspg2) and collagen Col13a1 (Wolf et al., 2009; Huang et al., 2011). Accordingly, conditional depletion of Fn1 in the ectoderm blocks lens placode invagination (Huang et al., 2011). Early studies of optic cup morphogenesis also identified cytoplasmic processes between the invaginating lens placode and the OV; however, the nature and function of these processes were not clear (McAvoy, 1980). Recent studies employing a set of markers for interepithelial processes, including F-actin, tubulins and keratin 18, identified these structures as filopodia, which are dynamic F-actin-based cellular protrusions that mostly originate in the lens pit and make contacts with the basal lamina of the retinal neuroepithelium (Chauhan et al., 2009). These filopodia serve as physical tethers that coordinate the reciprocal invagination process by controlling lens pit curvature (Fig. 3C-E) through actin-myosin contractile activity (Chauhan et al., 2009). They form at ∼E9.5 and are retracted by E11.5, once invagination is accomplished.

The invagination process also involves the elongation of columnar placodal cells concomitant with a change from cylindrical to conical shape mediated by apical constriction (Sawyer et al., 2010). The molecular pathway that controls apical constriction and its contribution to lens pit shape was investigated in a series of studies that analyzed conditional mutations in genes involved in actin remodeling, as well as changes in cell shape and the localization of cytoskeleton protein complexes during lens pit formation. These studies revealed that the cytoskeletal protein Shroom3 and the small Rho GTPase family members RhoA and Rac1 regulate apical constriction and placodal cell elongation during lens invagination (Plageman et al., 2010; Chauhan et al., 2011). In addition, activation of RhoA through the guanine nucleotide exchange factor Trio activates Shroom3 (Plageman et al., 2011). Most recently, the authors demonstrated the interaction of Shroom3 with the adherens junction protein p120-catenin (δ1-catenin) (Lang et al., 2014). Taken together, this interaction facilitates the recruitment of Shroom3 to adherens junctions, where it controls cell shape within the invaginating lens placode. It is noteworthy that, despite the documented importance of the above cytoskeletal and junctional proteins for cell shape, their loss did not prevent the initial invagination of the lens placode into the lens pit (Fig. 3C-E). Thus, additional mechanisms contribute to the initiation of the invagination process, such as the force generated by the basal filopodia described above (Chauhan et al., 2009). Apoptosis also plays a role in this process, and it was shown that the survival of the invaginating lens placodal cells is dependent on the functions of Six3 (Liu et al., 2006) and the evolutionarily conserved regulatory protein Mab21l1 (Yamada et al., 2003).

Disruptions to the processes that regulate lens placode invagination and lens vesicle formation can lead to two types of lens abnormalities. The first involves the formation of a corneal-lenticular stalk (called Peters' anomaly in humans, see Box 3). For example, the transcription factor Sip1 (Zeb2) is required for the separation of lens progenitors from corneal precursors as well as for the subsequent differentiation of secondary fibers: deletion of *Sip1* in the presumptive lens ectoderm led to the persistence of a lens stalk (Yoshimoto et al., 2005). The early activities of Sip1 are mediated by the activation of Foxe3 (Yoshimoto et al., 2005), whereas during secondary fiber differentiation Sip1 is required for inhibition of the surface ectoderm/corneal markers via a Foxe3-independent mechanism (Manthey et al., 2014). The second abnormality includes programmed cell death within the lens vesicle. In *Pitx3^−/−^* mouse embryos, for example, massive apoptosis in the lens vesicle reduces its size, and the inability of the residual cells to differentiate into lens fibers ultimately leads to the disappearance of the lens (*aphakia*) (Semina et al., 1997; Medina-Martinez et al., 2009).

Box 3. **Peters' anomaly**

Peters' anomaly is a rare genetic disease characterized by a persisting stalk between the nascent lens vesicle and the surface ectoderm that severely obstructs vision because of corneal opacification. The cellular mechanism responsible for this defect is inhibition of apoptosis in the stalk connecting the lens vesicle with the ectoderm (Pontoriero et al., 2009). Peters' anomaly is found in one-third of mouse *Pax6*^+/−^ embryos (Baulmann et al., 2002). In humans, Peters' anomaly is typically caused by missense mutations in a single allele of *PAX6* (see Cvekl and Tamm, 2004). Loss of both alleles of *AP-2α* (*Tfap2a*), *Cited2*, *Foxe3*, *Sox11* and compound inactivation of RXRα and RARγ nuclear receptors (*Rxra*^−/−^*;* *Rarg*^−/−^) produce similar defects (Lohnes et al., 1994; Medina-Martinez et al., 2005; Chen et al., 2008a; Pontoriero et al., 2008; Wurm et al., 2008). In addition, both Jag1 and Rbpj, which are components of Notch signaling, are required for proper separation of the lens vesicle from the surface ectoderm (Le et al., 2012). Notably, many of these genes, including *Cited2* (Chen et al., 2008a), *Foxe3* (Blixt et al., 2007) and *Sox11* (Wurm et al., 2008; Shaham et al., 2013), are directly or indirectly regulated by Pax6. Mutations in genes in this group are excellent candidates to explain abnormalities in anterior segment development. However, it remains to be determined how the Pax6-, Notch- and RA-dependent GRN regulates individual components of the programmed cell death machinery within cells of the lens-corneal stalk.

## Cell cycle exit and the initiation of lens cell differentiation

The 3D lens vesicle is a polarized structure. This polarization is induced by gradients of growth factors, including FGFs and BMPs, that are produced by the optic cup neuroepithelium as well as the prospective iris and ciliary body (Fig. 1) and regulate the pattern of differentiation across the developing lens. Anterior lens vesicle cells differentiate into the lens epithelium, which is a cuboidal sheet of cells that contains regions of very low, moderate and increased proliferative index (Zhou et al., 2006; Kallifatidis et al., 2011). Following cell cycle exit, posterior cells differentiate as highly elongated primary lens fibers that fill the lumen of the lens vesicle (Fig. 3F,G).

As in other cell types, lens cell proliferation is regulated via complexes between ‘pocket' family proteins [Rb1, p107 (Rbl1) and p130 (Rbl2)] and E2F proteins (E2F1-5), cyclins, cyclin-specific kinases and their negative regulators (p27^Kip1^ and p57^Kip2^) (see Griep, 2006). Somatic inactivation of *Rb1* completely disrupted lens fiber differentiation; the bulk of the lens was composed of proliferating cells, with many of them exhibiting p53-dependent programmed cell death (Morgenbesser et al., 1994). Moreover, conditional inactivation of *p53* in the lens resulted in the accumulation of proliferating, undifferentiated cells in the lens fiber cell compartment (Wiley et al., 2011). Overexpression of E2F1 or E2F2 in postmitotic transgenic lenses prompted re-entry into the cell cycle (Chen et al., 2000). The cell cycle regulators p27^Kip1^ and p57^Kip2^ are inhibitors of cyclin-dependent kinases and are expressed throughout the lens in distinct but overlapping patterns in the lens equatorial region. Depletion of p27^Kip1^ and p57^Kip2^ proteins completely disrupted the cell cycle exit of posterior lens vesicle cells, with notable differences to *Rb1^−/−^* lenses (Zhang et al., 1998).

The current challenge is to identify a direct link between the external differentiation signals (e.g. FGFs and BMPs) and the spatiotemporally restricted expression of p27^Kip1^ and p57^Kip2^, for example, by identifying the transcription factors that regulate the expression of these cell cycle regulators in the lens. These factors could work in various ways: via their upregulation in the posterior part of the lens vesicle followed, at later stages of development, by their increased expression in the lens transitional zone; via regionally specific post-translational modifications (e.g. phosphorylation, sumoylation and acetylation); or via signal-regulated removal of co-repressor proteins (see Barolo and Posakony, 2002). These models are not mutually exclusive. At present, a number of such candidate transcription factors have been identified. Gata3 (Maeda et al., 2009) and Prox1 (Duncan et al., 2002) are known to be highly expressed in only the posterior cells of the lens vesicle. In support of a role for these factors in lens cell proliferation, dysregulated cell cycle exit was observed in *Gata3* (Maeda et al., 2009), *Prox1* (Wigle et al., 1999), *Pitx3* (Medina-Martinez et al., 2009) and *Pax6* (Shaham et al., 2009) mutant lenses.

Analysis of the transition between proliferation and differentiation of primary lens fiber cells (Jarrin et al., 2012) showed that a balance of BMP and FGF signals regulates cell cycle exit. BMP activity, evaluated via its antagonist noggin, promotes FGF-dependent cell cycle exit. By contrast, FGF activity is required for proliferation and cell cycle exit, but is not sufficient to induce cell cycle exit (Jarrin et al., 2012). Finally, studies of the Bmpr1b (Alk6) receptor in the lens pointed to an asymmetry in primary lens fiber cell formation, with early differentiation occurring on the temporal side followed by delayed differentiation on the nasal side of the lens vesicle (Faber et al., 2002).

Notch signaling also regulates cell cycle exit in the lens. Studies involving inactivation of the of Notch ligand Jag1 (Le et al., 2009), the Notch2 receptor (Saravanamuthu et al., 2012) or the DNA-binding factor Rbpj (Jia et al., 2007; Rowan et al., 2008) established a role for Notch signaling in the lens. The expression of cyclins D1 and D2 and p27^Kip1^ was perturbed in *Rbpj* mutants (Rowan et al., 2008), while the Notch pathway effector protein Hey1 (Herp2) directly suppressed expression of p57^Kip2^ (Jia et al., 2007). The role of Wnt in lens cell proliferation remains questionable. Ectopic activation of Wnt/β-catenin signaling in the lens prevented cell cycle exit and lens fiber cell differentiation (Antosova et al., 2013; Shaham et al., 2009); nevertheless, the physiological significance of these gain-of-function studies remains to be determined.

## Differentiation of the lens epithelium

As the primary lens fibers initiate their elongation, the anterior cells of the lens vesicle differentiate into the lens epithelium (see Martinez and de Iongh, 2010). The lens epithelium contains stem/progenitor-like cells, which are the source of future fiber cells, and thus serves as an excellent model to study the regulation of lens growth and size. It has been shown that the ESC pluripotency factor Sox2, which is expressed during the early stages of lens formation (see above), becomes re-expressed in a specific population of adult lens epithelial cells and is required for their self-renewal (Arnold et al., 2011). Thus, the adult lens epithelium contains bona fide adult Sox2^+^ stem/progenitor cells (Arnold et al., 2011). Further characterization of these cells within their encapsulated niche as well as studies of Wnt signaling in lens epithelium (Cain et al., 2008) will be useful to understand the pathways and mechanisms that control continuous lens growth (Box 1). Of particular interest is the Hippo-Yap signaling pathway and its control of organ size (see Zhao et al., 2010). Yap is a multifunctional protein that is regulated at the level of phosphorylation and subcellular localization and is expressed in the lens epithelium as well as in the transitional zone. It has been shown that attenuated Hippo-Yap signaling via depletion of Yap from the lens vesicle stage reduces the pool of lens epithelial cells as a result of their premature differentiation (Song et al., 2014). It remains to be determined how Hippo-Yap signaling plays a role in lens stem/progenitor cell biology.

In addition to containing the stem/progenitor cell pool, the lens epithelium provides support to the fiber cell compartments through its connection with the aqueous humor, which fills the anterior chamber of the eye (Fig. 1). Apical membranes of the lens epithelium face the fiber cells, and basolateral membranes contact the aqueous humor (Fig. 3H). Among multiple candidate proteins, lens epithelial integrity is mediated by E- and N-cadherins and β-catenin (Pontoriero et al., 2009). The early expression of cadherins is regulated by AP-2α (Tfap2a) (Pontoriero et al., 2008), and loss of AP-2α after the formation of the lens vesicle results in the formation of an abnormal multilayered lens epithelium (Kerr et al., 2014). As age-onset cataract is characterized by disrupted lens epithelium function, including epithelial cell loss (see Petrash, 2013), the continuation of studies related to lens epithelial homeostasis is crucial for understanding cataractogenesis.

## Lens fiber cell differentiation and development

Lens fiber cell differentiation is characterized by the following processes, many of which evolved to minimize light scattering to ensure transparency: (1) crystallins accumulate in lens fibers, giving the lenss its transparency and refractive power (Bassnett et al., 2011); (2) cellular elongation is accompanied by dramatic cytoskeletal remodeling, including the formation of a lens-specific intermediate filament cytoskeleton composed of Bfsp1 (filensin) and Bfsp2 (CP49) (Rao and Maddala, 2006; Fudge et al., 2011); (3) the maturing lens fibers degrade their intracellular organelles as their presence would compromise lens transparency (see Bassnett, 2009); (4) lens fibers establish multiple transport and cell-to-cell communication systems to receive and distribute water, ions and nutrients between the lens epithelium and the less metabolically active fiber cell compartment (Mathias et al., 2010), while centrally located fibers (in the lens ‘nucleus') undergo terminal differentiation, which includes the formation of interdigitized processes between individual hexagonal lens fibers and the eventual generation of a stratified syncytium (Shi et al., 2009). Below, we discuss these various aspects of fiber cell differentiation.

### Lens fiber cell differentiation: the reuse and inclusion of additional signaling pathways

A general model for secondary lens fiber differentiation is based on the action of the FGF (Turner and Grose, 2010), BMP (Massague et al., 2005) and Wnt (Groves and LaBonne, 2014) signaling pathways. Additional pathways, including the insulin-like growth factor receptor 1 (IGF1R)/NF-κB (Nfkb1), phosphatidylinositol 3-kinase (PI3K), MAPK/JNK-mTOR and mitochondrial cell death pathways, have also been identified to play specific roles in lens fiber cell formation.

Studies using primary lens cultures identified FGF2 (bFGF) as a concentration-dependent soluble factor capable of inducing lens fiber cell differentiation (Lovicu and McAvoy, 2005). Different concentrations of FGF2 elicited distinct cellular responses (McAvoy and Chamberlain, 1989). Half maximal activities for the three responses – proliferation, migration and differentiation – are 0.15, 3 and 40 ng/ml FGF2, respectively. The highest concentrations of FGF2 induce cellular elongation and other attributes of lens terminal differentiation, including the accumulation of crystallins and the degradation of subcellular organelles. The systematic deletion of the mouse FGF receptors *Fgfr1*, *Fgfr2* and *Fgfr3* has been conducted in postmitotic lens fibers (Zhao et al., 2008). Although lens vesicles were formed in the triple conditional knockout, a range of subsequent defects included abnormal cell proliferation, lack of cell elongation, and increased apoptosis, consistent with a multitude of roles for FGF signaling during lens development. Studies of adaptor/docking proteins, including Frs2α, Shp2 (Ptpn11), Gab1 and Gab2 (Gotoh et al., 2004; Madakashira et al., 2012; Li et al., 2014), Erk1/2 kinases, namely Erk2 (Mapk1) (Upadhya et al., 2013), and enzymes responsible for synthesis of the heparan sulfate proteoglycans (HSPGs) (Pan et al., 2006; Qu et al., 2011), further supported a model in which an Frs2α-Shp2 complex is a key mediator of FGF/MAPK signaling in the lens.

Like FGF signaling, BMP signaling also controls secondary lens fiber cell differentiation. BMP4 and BMP7 are endogenously expressed by lens cells (Boswell et al., 2008). Abolishing BMP2/4/7 signaling using noggin prevented FGF from inducing fiber cell-specific protein expression (Boswell et al., 2008). External FGF2 induced the endogenous expression of BMP2 and BMP4 and of the ‘canonical' target genes of BMP signaling, *Id1* and *Id3* (Kowanetz et al., 2004), in rat lens explants (Wolf et al., 2013a).

Dramatic reorganization of the actin cytoskeleton from stress fibers to cortical fibers, as well as additional remodeling of the spectrin membrane cytoskeleton, are hallmarks of the early stages of lens fiber cell differentiation (Lee et al., 2001; Weber and Menko, 2006a). It has been proposed that lens cytoskeletal remodeling requires caspase-dependent proteolysis of α- and β-spectrins (Lee et al., 2001), and this model is further supported by the identification of caspase-3-like activity in the equatorial lens epithelium (Weber and Menko, 2005). Histological analysis of *Casp3^−/−^;* *Casp6^−/−^* lenses revealed relatively normal tissue organization (Zandy et al., 2005). Thus, the proteolytic mechanisms that regulate lens differentiation are likely to employ additional enzymes, such as the VEIDase (caspase-6-like) (Foley et al., 2004; Zandy and Bassnett, 2007), and further studies will be required to clarify this issue.

Regulation of the lens cytoskeleton has also been linked to multiple signaling processes involving cadherins (Leonard et al., 2013), integrins (Basu et al., 2014a), IGF1R/NF-κB (Basu et al., 2012), PI3K (Weber and Menko, 2006b) and the mitochondrial cell death pathway (Weber and Menko, 2005; Basu et al., 2012). PI3K is a downstream component of FGF signaling that mediates cell survival in multiple ways, via inhibition of caspase-9, BAD and BAX proteins, and by another serine-threonine kinase, AKT (Goetz and Mohammadi, 2013). Pharmacological inhibition of PI3K blocked the initiation of lens fiber cell differentiation via attenuation of the small Rho GTPase Rac1 (Weber and Menko, 2006b). Rac1 has been shown to control lens actin cytoskeletal dynamics, survival, migration and lens shape (Maddala et al., 2011). Collectively, these studies reiterate that early lens cells are prone to apoptosis, and that the pro-survival functions of FGF and IGF1R are essential for lens development.

### Regulation of crystallin gene expression by FGF, c-Maf and Pax6

**Fig. 5.**
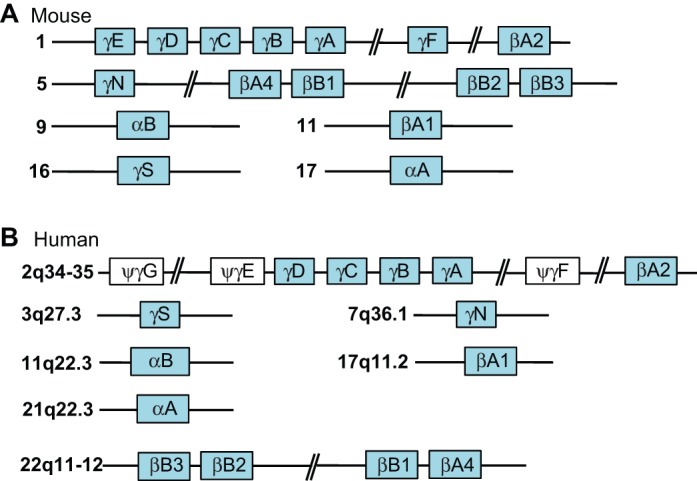
**Crystallin genes in the mouse and human genomes.** (A) Crystallin genes in mouse. (B) Crystallin genes and pseudogenes in human. The vertebrate crystallin genes were formed by multiple duplications of the ancestral small heat shock protein (α-crystallins) and the founding member of the β/γ-crystallin gene family. Gene duplications and functional similarities between individual α- and β/γ-crystallins, coupled with some very unique roles [e.g. those played by βA3/A1- and βB2-crystallins (Sun et al., 2013; Valapala et al., 2014)], impede eye and lens research, as both the serial inactivation of each β/γ-crystallin gene and the generation of a large number of compound mutants are challenging tasks. An alternate approach is to generate large-scale deletions in mouse chromosomes 1 and 5 (see A) using a Cre/loxP or a CRISPR-Cas9 system, followed by gene ‘rescue' experiments by reintroducing individual and/or combined crystallin gene transgenic units. The individual and compound mutants of *Cryaa* and *Cryab* genes have already provided invaluable insights into the detailed aspects of lens morphogenesis (Brady et al., 1997; Morozov and Wawrousek, 2006; Xi et al., 2006).

Lens fiber cell differentiation is also characterized by the expression and accumulation of crystallins, which serve as structural proteins and in some cases, such as α-crystallins, are also powerful inhibitors of apoptosis in lens fiber cells (Morozov and Wawrousek, 2006). The mouse genome contains 16 crystallin genes localized on six chromosomes (Fig. 5A). The human crystallin gene family is smaller as it includes three pseudogenes (Fig. 5B). The expression of individual crystallin genes is temporally and spatially regulated in the lens (see Piatigorsky, 1981). In mice, the expression of αB-crystallin initiates in the lens placode (E9.5) and αA-crystallin expression commences later in the invaginating lens pit (E10.5-E11). RNA- and protein-based studies of α-crystallins indicate an absence of post-transcriptional control. By contrast, expression studies of β/γ-crystallin genes (e.g. high-density oligonucleotide hybridizations and RNA-seq) identified their transcripts in the lens placode, lens pit and lens vesicle (Huang et al., 2011; Lachke et al., 2012; Wolf et al., 2013b). Since the β/γ-crystallin proteins are detected in the differentiating lens fibers, the gap between the RNA and the protein production data points to translational control, as supported by recent studies of the translational initiation factor Eif3h in zebrafish embryos, which regulates a defined subset of transcripts including the crystallins during lens development (Choudhuri et al., 2013).

**Fig. 6.**
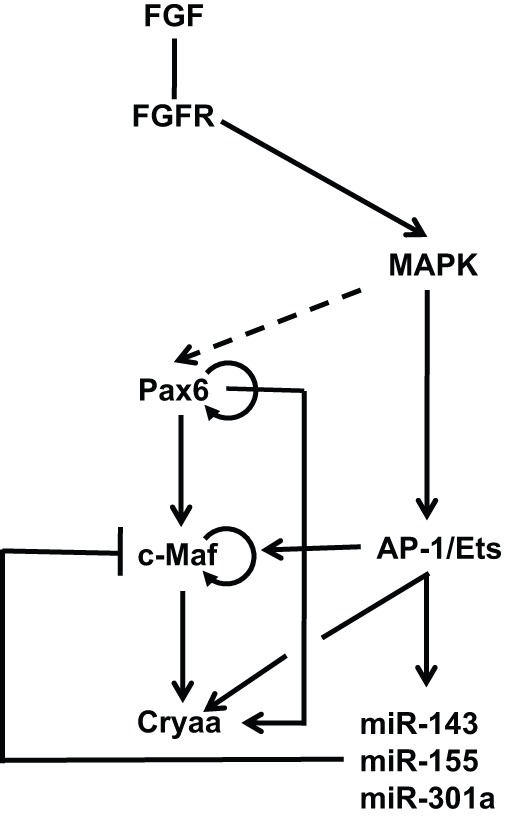
**Gene regulatory networks that control crystallin gene expression.** The ‘core' GRN for crystallin gene expression comprises Pax6, c-Maf and individual crystallins and functions through a feed-forward loop that involves Pax6 and c-Maf autoregulation. Our recent studies established a direct link between FGF signaling and crystallin gene expression mediated by AP-1 (e.g. c-Jun) and Ets transcription factors. A negative-feedback mechanism modulates the expression of c-Maf via multiple miRNAs that recognize its 3′-untranslated region. FGF signaling directly controls the transcription of αA-crystallin (*Cryaa*) via the DCR1 5′-enhancer. It remains to be determined if ERKs/MAPKs directly phosphorylate Pax6 proteins (dashed arrow) at the level of the crystallin GRN as suggested by multiple studies (Mikkola et al., 1999; Yoo et al., 2011), to examine the regulatory role of protein phosphatase 1 in this process (Yan et al., 2007), and to determine whether other crystallin genes contain similar arrays of AP-1/Ets sites in their promoters/enhancers.

The transcription factors c-Maf, Hsf4, Pax6, Pitx3, Prox1, RARβ/RXRγ, Sox1 and Sox2 serve as common regulatory transcription factors that are directly employed by two or more crystallin genes (Cvekl and Duncan, 2007). Analysis of *c-Maf* null lenses identified a significant reduction in the expression of all crystallins examined when compared with wild-type lenses (Kawauchi et al., 1999; Kim et al., 1999; Ring et al., 2000). Expression of c-Maf in the lens is controlled by a Pax6-regulated distal enhancer (Xie and Cvekl, 2011). Taken together, Pax6, c-Maf and crystallins form a feed-forward loop with two autoregulatory circuits involving Pax6 and c-Maf. As FGF signaling controls *c-Maf* expression via its promoter (Q. Xie, R. S. McGreal, L. W. Reneker, L. S. Musil and A.C., unpublished), the expression of crystallin genes regulated by c-Maf is thus linked to the FGF signal transduction pathway (Fig. 6).

### Elongation and alignment of lens cell fibers

Elongated lens fibers are characterized by a number of morphological features that evolved to minimize light scattering, to permit cell-to-cell communication, to generate lens mechanical stiffness and elasticity for focusing and accommodation, and to compensate for the lack of intracellular organelles (Bassnett et al., 2011). Elongation of a lens fiber cell continues until its apical and basal ends terminate at the poles of the lens (Fig. 3H). The precise alignment and orientation of fibers generates lens-characteristic suture lines (Fig. 1) (Kuszak et al., 2004).

Lens morphogenesis therefore depends both on the execution of the differentiation program and on the correct assembly of the epithelial and fiber cells into the 3D functional structure. Recent studies exposed a key role for the Wnt/planar cell polarity (PCP) pathway in directing lens fiber morphology (Sugiyama et al., 2010). This conclusion is based on the findings that each lens fiber cell has a primary cilium on its hexagonal apical surface that is polarized towards the side that faces the anterior pole, and that inactivation of genes encoding components of the PCP pathway (e.g. *Rac1*, *Vangl2* and *Celsr1*) disrupts cilium orientation and lens fiber morphology (Sugiyama et al., 2010, 2011). Overexpression of secreted frizzled-related protein 2 (Sfrp2), which is a negative regulator of Wnt signaling, disrupted the elongation of the tips of lens fibers towards the lens poles (Chen et al., 2008b). A similar phenotype was found in abl-interactor 2 (*Abi2*) mutant lenses (Grove et al., 2004), raising the possibility that Abi2 is a downstream target of Wnt/PCP signaling (Sugiyama et al., 2011). More recently, *in vitro* studies suggest that epithelial-derived Wnt directs the alignment and orientation of the lens fibers by triggering the PCP pathway and translocation of frizzled and the centrosome to the apical tip of the elongating fiber cell (Dawes et al., 2013, 2014).

The establishment of lens fiber apical-basal polarity is further regulated by the atypical Ser/Thr protein kinase C (aPKCλ, or PRKCι) (Sugiyama et al., 2009). aPKCλ binds to β-catenin, a regulator of cell-cell contact and Wnt signal transduction, and forms a complex with the polarity proteins Par3 and Par6. Conditional deletion of two other polarity proteins, discs large 1 (Dlg1) and Scribble (Scrib), in the lens was used to probe the link between epithelial cell polarity and differentiation. *Dlg1* mutant lenses also exhibited abnormal lens fiber cell alignment and curvature (Rivera et al., 2009). The *Scrib* mutant lenses showed multiple defects in lens morphogenesis, disrupted lens fibers, and vacuoles in the fiber cell compartment (Yamben et al., 2013).

Ephrin receptors, which control a multitude of processes during embryonic development, are also important for lens fiber cell elongation and normal lens development. In *Epha2^−/−^* lenses, equatorial epithelial cells do not form meridional rows, nor do they form the lens fulcrum (Fig. 3H) (Cheng et al., 2013). In *Epha5^−/−^* lenses, lens fiber cells are rounded and irregular in cross-section. The EphA2 receptor regulates the adherens junction complex by stimulating the recruitment of β-catenin to membrane-associated N-cadherin (Cooper et al., 2008). Collectively, *Epha2* and *Epha5* mutations produce disorganized lens cells with altered refractive indices and cataracts (Cooper et al., 2008; Cheng et al., 2013).

Lens fiber cells also form a specialized intermediate filament cytoskeleton composed of two fiber cell-specific proteins, Bfsp1 and Bfsp2 (Fudge et al., 2011). These proteins form structures known as beaded filaments that change their localization from membrane bound to more cytoplasmic following organelle degradation; Bfsp2 synergizes with tropomodulin 1 (Tmod1) to control lens mechanical stiffness through synergism between the spectrin-actin membrane skeleton and the beaded filament cytoskeleton (Gokhin et al., 2012).

Lens fiber morphogenesis also requires expression of the highly abundant major intrinsic protein (MIP, or aquaporin-0), which is a water channel and a major lens membrane structural protein (Agre and Kozono, 2003), and the connexins Cx43 (Gja1), Cx46 (Gja3) and Cx50 (Gja8), to establish gap junction channels (see Mathias et al., 2010). The importance of MIP and connexins is exemplified by the number of mutations found in these genes in mouse and human that result in congenital cataracts (Shiels and Bassnett, 1996). Deletion of the *Mip* gene in mice disrupts the formation of sutures, which are required for maintenance of the lens fiber architecture, resulting in perturbed accommodation and focus properties of the ocular lens (Shiels et al., 2001). The expression of MIP in lens is regulated by a combination of FGF/MAPK and JNK signaling (Golestaneh et al., 2004) and by Pitx3 (Sorokina et al., 2011). Following organelle degradation and tissue remodeling (as discussed below), lens fiber cell membranes form ball-and-socket structures in the deep cortex. Following elongation, a stratified syncytium forms that is hypothesized to play a major role in establishing a uniform refractive index within any one stratum (Shi et al., 2009).

### Organelle degradation in lens fibers

The presence of subcellular organelles interferes with lens transparency. Thus, the lens employs autophagy and related processes (see Box 4) to generate an organelle-free zone (OFZ) throughout the region comprising the optic axis (Fig. 1). The morphology of lens fiber cell nuclei also changes (e.g. due to chromatin condensation) at least 48 h prior to their disappearance (Counis et al., 1998). Many of these features are similar to the nuclear changes that occur in cells undergoing the initial phases of apoptosis. However, lens fiber cells are preserved and, if any apoptosis-like processes occur in the lens fibers, they are ultimately suppressed (Bassnett and Mataic, 1997).

Box 4. **Organelle degradation: autophagy, mitophagy and nucleophagy**

A hallmark of lens fiber cell differentiation is the highly organized degradation of intracellular organelles, including the endoplasmic reticulum, Golgi apparatus, mitochondria and nuclei, to generate a lens organelle-free zone (OFZ) (Fig. 1). Retention of these organelles would otherwise compromise lens transparency. Other than the lens, degradation of nuclei is found only in mammalian erythrocytes and skin keratinocytes. Erythrocytes are enucleated through the action of macrophages that engulf the cell and extrude its nucleus, and this is followed by nuclear degradation (Yoshida et al., 2005). In addition, erythrocyte mitochondria are also completely degraded through mitophagy (Sandoval et al., 2008). By contrast, skin keratinocytes ‘slowly’ lose their nuclei via caspase-independent ‘cornification’ (Lippens et al., 2009). Although red blood cells and keratinocytes are turned over, organelle-free lens fiber cells have to be maintained throughout life. The molecular mechanisms of lens fiber nuclear degradation are poorly understood, although its has recently been shown that mitophagy degrades mitochondria (Costello et al., 2013), and that autophagy is involved in nuclear degradation (Basu et al., 2014b). Mutations in FYVE and coiled-coil domain containing 1 protein (*FYCO1*) cause human congenital cataracts (Chen et al., 2011) and this has implicated autophagy in lens formation and/or function. Accordingly, the vertebrate lens expresses a range of autophagy-mitophagy genes and proteins in a differentiation-specific manner (Brennan et al., 2012; Basu et al., 2014a,b; Chauss et al., 2014).

The molecular basis for lens fiber cell denucleation appears to be complex, and the disruption of genes encoding multiple classes of proteins results in nuclear retention in the presumptive OFZ. These protein classes include apoptosis regulatory proteins [p53 (Wiley et al., 2011)], cell cycle regulatory proteins [Cdk1 (Chaffee et al., 2014)], chromatin remodeling enzymes [Brg1 (Smarca4) and Snf2h (Smarca5) (He et al., 2010; S. He and A.C., unpublished)], transcription factors [Gata3, Foxe3 and Hsf4 (Fujimoto et al., 2004; Medina-Martinez et al., 2005; Maeda et al., 2009)], α- and γ-crystallins (Sandilands et al., 2002; Graw et al., 2004; Wang et al., 2007; Gupta et al., 2011), DNA repair and associated proteins [Ddb1, Nbs1 (Nbn) and Ncoa6 (Cang et al., 2006; Yang et al., 2006b; Wang et al., 2010)], DNA endonucleases [DNase IIβ (Nishimoto et al., 2003)] and lipoxygenase pathway enzymes [Alox15 (van Leyen et al., 1998)]. Pharmacological manipulation of autophagy by inhibiting MAPK/JNK-mTORC1 signaling in chicken lenses was shown to disrupt the denucleation process (Basu et al., 2014b). Although *Jnk1^−/−^;Jnk2^−/−^* (*Mapk8^−/−^;Mapk9^−/−^*) mouse eyes show retinal and lens abnormalities (Weston et al., 2003), organelle degradation in the mutated lens fibers remains to be analyzed. Furthermore, the link between lens fiber cell differentiation and organelle degradation can be mediated by the FGF/PI3K signaling arm (Webb and Brunet, 2014) described above; however, further studies are needed to clarify this mechanism in the lens.

## Conclusions

Significant progress in understanding the cellular and molecular mechanisms of lens morphogenesis has been accomplished in recent decades. The driving force behind discoveries of lens regulatory genes was a combination of molecular cloning based on sequence homologies between genes that control *Drosophila* and mouse eye formation (Oliver and Gruss, 1997; Donner and Maas, 2004), genetic studies of human congenital eye malformations (Hever et al., 2006; Williamson and FitzPatrick, 2014), and the utilization of both ‘classical' mouse models, such as *small eye* (*Sey*), *aphakia* (*ak*) and *dysgenetic lens* (*dyl*) (see Box 5), and the generation of novel mutants (Graw, 2009). By contrast, lens structural proteins were mostly identified through protein purification and comprehensive proteomic approaches (Lampi et al., 2002; Greiling et al., 2009; Wang et al., 2013). Rapid advances in DNA and RNA sequencing methods, as well as the mapping of protein-DNA (ChIP-seq) and protein-RNA (HITS-CLIP) complexes, will greatly aid the identification and functional analysis of novel lens transcripts and their splice variants, and of short and long non-coding RNAs (e.g. miRNAs and lncRNAs). Likewise, the analysis of proteins, their post-translational modifications in normal and pathological conditions, coupled with the development of highly specific antibodies targeted to recognize different forms and states of proteins, including antibodies that work inside living cells (Attreed et al., 2012), will play pivotal roles in deciphering the molecular mechanisms of lens development.

Box 5. ***Small eye* and cataractogenesis**

The *small eye* (*Sey*) phenotype is a recurrent lens abnormality found in laboratory mouse and rat models that is characterized by lenses of a reduced size that are prone to cataractogenesis. The corresponding human condition is aniridia (Hever et al., 2006; Williamson and FitzPatrick, 2014). The classical *Sey* allele (Hogan et al., 1988) harbors a mutated *Pax6* gene (Shaham et al., 2012). The reduced size of the lens in mice can be caused by smaller lens placodes, as in the case of *Pax6* haploinsufficiency, which yields approximately half the normal number of the lens progenitor cells that build the lens placode (van Raamsdonk and Tilghman, 2000). Inactivation of Meis1, an upstream regulator of Pax6 (Zhang et al., 2002), also results in small lenses (Hisa et al., 2004). In addition, reduced proliferation of the cells comprising the lens vesicle, concomitant with a smaller lens, was found following inactivation of a group of growth control genes, including *c-Myc* (Cavalheiro et al., 2014), *Msx2* (Zhao et al., 2012) and *Nf1* (Carbe and Zhang, 2011). Congenital human cataracts have been reported in 50-85% of aniridia cases and these cataracts develop from smaller anterior or posterior opacities that are already present at birth (see Cvekl and Tamm, 2004).

Given the prominent roles of Pax6 in non-lens tissues, it is necessary to identify the precise molecular mechanisms by which Pax6 activates the lens developmental program and suppresses alternative programs, particularly to identify the tissue-specific co-factors that function with Pax6 in the context of the lens, as recently recognized for the RPE (Raviv et al., 2014). Recent studies have suggested that the Pax6-mediated recruitment of distinct chromatin remodeling enzymes, such as components of the SWI/SNF complex, can elicit different transcriptional outputs (Yang et al., 2006a; Ninkovic et al., 2013; Tuoc et al., 2013). Furthermore, and in addition to Brg1 and CBP/p300, inactivation of genes encoding the chromatin remodeling proteins BCOR (Ng et al., 2004), Cited2 (Chen et al., 2008a), Med1 (PBP) (Crawford et al., 2002) and Kdm5b (Jarid1b) (Albert et al., 2013), disrupts lens development. Studies in zebrafish have also revealed roles for DNA methylation in embryonic eye formation, including in lens development (Rai et al., 2006, 2010), although studies of DNA methylation during mammalian lens development and in the adult lens epithelium are still in their infancy (Tittle et al., 2011; Seritrakul and Gross, 2014).

Advances in sequencing technologies combined with functional studies have exposed the prevalence and importance of miRNAs as important regulators of tissue differentiation, including the eye (Conte et al., 2013). The importance of miRNAs for lens development was shown by lens-specific depletion of Dicer1, an RNase III type enzyme that is essential for the biogenesis of most miRNAs. *Dicer1* deletion in the PLE did not prevent formation of the lens vesicle but did result in progressive lens dystrophy due to reduced proliferation and increased cell death (Li and Piatigorsky, 2009). Although these findings suggest that miRNAs are not essential for early stages of lens morphogenesis, there is a possibility that some miRNAs are stable and could persist for several days after Dicer1 inactivation. Inactivation of Dicer1 during secondary fiber differentiation produced degenerated lenses, and the markedly altered miRNA profiles in response to activated FGF signaling in lens cell culture systems further substantiate key cell-autonomous roles for miRNAs in the maintenance of the lens epithelium compartment and for the differentiation of lens fiber cells (Wolf et al., 2013a). The specific roles of individual miRNAs in the lens require comprehensive functional studies of their multiple predicted target mRNAs. The most extensively investigated miRNA in lens development is miR-204. In the medaka fish, miR-204 is required for lens and retina development via regulation of Meis2, which in turn modulates Pax6 expression (Conte et al., 2010). It was recently established in mouse that Pax6 regulates the expression of *Mir204*, which is embedded in the *Trpm3* locus, in the lens, retina and distal optic cup (Shaham et al., 2013). In the lens, miR-204 partially mediates Pax6 activity in maintaining lens fate, preventing cell migration, and in controlling the level of expression of factors required in lens fibers (Avellino et al., 2013; Shaham et al., 2013). Considering the prevalence of miR-184 in the lens and its inducibility by FGF2 (Wolf et al., 2013a,b), it is not surprising that mutations in the miR-184 seed region were identified in families with familial keratoconus and cataract (Hughes et al., 2011).

Another level of gene regulation involves control of RNA translation by RNA-binding proteins. The RNA-binding protein Tdrd7 is highly expressed in lens fiber cells and regulates the post-transcriptional control of transcripts encoding important lens regulatory proteins (Lachke et al., 2011). We propose that miRNAs and RNA-binding proteins represent promising new research areas to better understand lens development and disease.

The architecture of lens-specific GRNs, including those involving AP-2α, c-Maf, Gata3, Hsf4, Pitx3, Prox1 and Sox1, remain to be established, for example via a combination of ChIP-seq and RNA-seq studies. Emerging studies have now identified direct regulation of *Foxe3* by Pitx3 (Ahmad et al., 2013) and multiple direct targets of Hsf4 in the lens, such as DNase IIβ and FGFRs (Fujimoto et al., 2008). Equally important are studies of signal-regulated factors (including c-Jun, CREB, Ets, Rbpj, Smad and Sp1) to reconstruct GRNs of lens induction, exit from the cell cycle, differentiation, and organelle degradation. These studies will help reveal additional regulatory mechanisms of lens differentiation, such as post-translational modifications (Gong et al., 2014), and will aid the ongoing identification of novel lens disease-associated genes (Lachke et al., 2012).

Last, but not least, lens research will have an impact on other fields, including comprehensive studies of crystallins as negative regulators of apoptosis, analysis of the modulatory proteins of autophagy, and the identification of dysregulated genes in cancer and neurodegenerative diseases. Advances in lens research will also aid our general understanding of signal transduction specificity, growth factors, mechanisms of cell cycle exit, chromatin remodeling, nuclear biology and eye evolution. For example, αB-crystallin plays a major role in the pathology of multiple sclerosis (Ousman et al., 2007), neuroinflammation (Shao et al., 2013) and cardiac myopathy (Bhuiyan et al., 2013). Similarly, Pax6-dependent expression of αA-crystallin in olfactory bulb neurons protects them from cell death (Ninkovic et al., 2010). Activity of the argonaute 2 (Ago2) protein, which is a catalytic subunit of the RNA-induced silencing complex, is also regulated by αB-crystallin (Neppl et al., 2014). Thus, functional studies of lens genes and proteins will provide novel data on their multifunctionality outside of their ‘home' lens tissue and might provide novel insights into a multitude of diseases.
